# Protein Quality Control at the Sarcomere: Titin Protection and Turnover and Implications for Disease Development

**DOI:** 10.3389/fphys.2022.914296

**Published:** 2022-06-30

**Authors:** Sebastian Kötter, Martina Krüger

**Affiliations:** Department of Cardiovascular Physiology, Medical Faculty and University Hospital Düsseldorf, Heinrich-Heine-University Düsseldorf, Düsseldorf, Germany

**Keywords:** connectin, degradation, proteasome, autophagy, proteases, heat-shock-proteins

## Abstract

Sarcomeres are mainly composed of filament and signaling proteins and are the smallest molecular units of muscle contraction and relaxation. The sarcomere protein titin serves as a molecular spring whose stiffness mediates myofilament extensibility in skeletal and cardiac muscle. Due to the enormous size of titin and its tight integration into the sarcomere, the incorporation and degradation of the titin filament is a highly complex task. The details of the molecular processes involved in titin turnover are not fully understood, but the involvement of different intracellular degradation mechanisms has recently been described. This review summarizes the current state of research with particular emphasis on the relationship between titin and protein quality control. We highlight the involvement of the proteasome, autophagy, heat shock proteins, and proteases in the protection and degradation of titin in heart and skeletal muscle. Because the fine-tuned balance of degradation and protein expression can be disrupted under pathological conditions, the review also provides an overview of previously known perturbations in protein quality control and discusses how these affect sarcomeric proteins, and titin in particular, in various disease states.

## 1 Introduction

The sarcomere scaffold of striated muscle cells is formed by three filaments: The thick filament consisting of myosin, the thin filament formed by actin, and the titin filament. While myosin and actin are mainly responsible for the Ca^2+^-dependent force development of the sarcomere, several different functions are attributed to the titin filament. Due to its very specific structure and position in the sarcomere, titin plays a particularly important role in shaping the viscoelastic properties of myofilaments. In this role, titin acts as a molecular spring and, along with collagen, is the main determinant of passive elastic force in muscle cells (Granzier and Irving, 1995; Linke, 2008; Tskhovrebova and Trinick, 2010; Gautel, 2011b). In addition, titin participates in the complex mechanisms determining length-dependent activation, is responsible for keeping thick filaments in the center of the sarcomere, assists in sarcomere assembly, and is a hotspot for various processes of mechano-chemical signal transduction (Krüger and Linke, 2011). The elastic properties of cardiac titin change dynamically during cardiac development and may also be pathologically altered during the course of cardiac disease (Linke and Hamdani, 2014). These changes in the titin molecule have a strong impact on diastolic function of the myocardium (Koser et al., 2019). The central position of titin in the sarcomere, coupled with its role as a signaling node allows it to function as an important mechanosensor (Krüger and Linke, 2011). Because the protein is subjected to high mechanical stress in the working sarcomere, the titin filament must be subject to tightly regulated quality control. However, in a sarcomere that is constantly contracting and relaxing, quality control of a giant protein like titin is a huge challenge. In this review, we provide a brief overview on the mechanisms of protein quality control (PQC) in the sarcomere with a particular focus on the mechanisms of titin degradation known to date. In addition, we compile some well-characterized pathologies in which PQC mechanisms are impaired, leading to a significant loss of myocyte function.

## 2 Titin—A Brief Overview on Structure and Function

The giant protein titin is encoded by a single gene with 364 exons resulting in a theoretical (maximum) size of 4.2 MDa (Bang et al., 2001). A single titin molecule spans the distance of 1 µm from the Z-disk to the M-line. Titin consists mainly of serially linked immunoglobulin-like domains (Ig domains), fibronectin type III domains (FN3 domains), and some unique sequences (us) (Bang et al., 2001). The NH_2_ terminus of titin is anchored to the sarcomeric Z-disk by interaction with nebulin or the cardiac isoform nebulette (Witt et al., 2006), *α*-actinin 2 (Sorimachi et al., 1997; Labeit et al., 2006), and telethonin (Mues et al., 1998; Granzier and Labeit, 2004; Miller et al., 2004; Lange et al., 2006). Telethonin connects the NH_2_-terminal parts of two titin molecules from one sarcomere in a palindromic manner (Zou et al., 2006). In a complex with the Z-disk component actinin and the muscle LIM protein (MLP), titin and telethonin form a mechanical stretch-sensing complex (Knöll et al., 2002). The I-band part of titin is not firmly attached to the adjacent filaments and can thus act like an elastic molecular spring that contributes to the passive stiffness of cardiac and skeletal muscles. It is composed of the proximal Ig domains, a heart-specific N2B domain (including the unique N2-B sequence, N2-bus), the middle Ig domains, the N2A domain (N2BA isoform), the PEVK domain [named for the high abundance of proline (P), glutamic acid (E), valine (V), and lysine (K) residues], and finally the distal Ig domains. With a size of ∼ 2 MDa, the A-band part of titin is the largest part of the molecule (Bang et al., 2001) and mainly consists of fibronectin type III and Ig-domains, organized in 7-domain and 11-domain super repeats, respectively (Labeit et al., 1992). Titin is tightly connected to myosin and myosin-binding protein C (Tskhovrebova and Trinick, 2004; Lange et al., 2006). The M-band portion of titin contains several inserted sequences and the titin-kinase-domain in the M-band periphery (Bang et al., 2001; Gautel, 2011a).

In cardiac muscle cells, titin occurs in two isoforms: the longer and more compliant N2BA isoform (∼3.2–3.3 MDa) and the shorter and more rigid N2B isoform (∼3.0 MDa). Skeletal muscle cells contain a third isoform type, the N2A isoform with the slightly longer muscle-specific splice variants (3.3–3.7 MDa) (Freiburg et al., 2000; Neagoe et al., 2003; Prado et al., 2005). In cardiac muscle cells the ratio of N2BA and N2B largely determines titin based passive stiffness (Krüger and Linke, 2011).

The composition of titin isoforms can be dynamically adjusted. For example, the composition of titin isoforms changes from a predominant fetal N2BA isoform to adult N2BA and N2B isoforms during cardiac development, which is enhanced by stimulation with thyroid hormone 3 or insulin through triggering the PI3-K/AKT/mTOR pathway (Krüger et al., 2008; Krüger et al., 2010). The ratio of N2BA:N2B in human cardiomyocytes is about 30%:70% (Neagoe, 2002). All titin isoforms are transcribed and translated from a single gene by alternative splicing, which is strongly regulated by the splicing factor RNA-binding motif 20 (RBM20) Silencing of RBM20 leads to the formation of a giant titin isoform (N2BA-G, 3.9 MDa) (Guo et al., 2012; Zhu et al., 2017). Splicing events occur mainly within the I-band portion of titin but can also involve the Z-disk and M-band (Krüger and Linke, 2011). In heart failure, severe deviations in the composition of titin isoforms may occur due to altered splicing behaviour. In this context, a shift towards an either elevated or decreased N2BA:N2B ratio has been observed in several systolic or diastolic heart failure models (Linke and Hamdani, 2014).

While changing isoform composition is a long-term mechanism, posttranslational modification of elastic I-band domains can affect titin-based stiffness more dynamically. The most intensively studied posttranslational modification is phosphorylation of elastic spring elements in the I-band part of titin (Linke and Hamdani, 2014). Phosphorylation of the cardiac N2-B-domain by Protein Kinase A (PKA) (Yamasaki, 2002), Protein Kinase G (PKG) (Krüger et al., 2009), Cam-Kinase IIδ and Extracellular regulated Kinase 1/2 (ERK1/2) lowers titin-based passive stiffness whereas phosphorylation of the PEVK domain by PKC*α* increases titin-based passive stiffness (Hidalgo et al., 2009). Alterations of the phosphorylation levels have been demonstrated for different human pathologies with elevation of PEVK and reduced N2B phosphorylation in DCM, HCM and diabetes type 2 (van Heerebeek et al., 2012; Hamdani et al., 2013; Kötter et al., 2013; Hopf et al., 2018) resulting in higher stiffness compared to healthy controls. Higher PEVK and lower N2B phosphorylation has also been observed in the remote myocardium of mice after 3 days of permanent LAD ligature (Kötter et al., 2016). Acute and chronic elevation of titin-based passive stiffness could be tantamount with higher mechanical strain, increased working load and subsequent abrasion potentially lowering titin filament lifetime. This could lead to the necessity of faster replacement and degradation of titin filaments.

Oxidative stress due to higher levels of reactive oxygen species (ROS) can result in oxidative modification of myofilament components, thereby affecting myocyte contractility. Oxidative modification of sarcomere proteins and especially titin has been reported for several conditions like ischemia/reperfusion, in heart failure or muscular dystrophies (Beckendorf and Linke, 2015). S-glutathionylation lowers titin-based passive stiffness, disulphide bonding increases it (Grützner et al., 2009; Alegre-Cebollada et al., 2014). Unfolded domain oxidation within Ig-domains of the I-band also affects titin elasticity (Loescher et al., 2020). Although most cysteines are buried within folded Ig-domains and can only be oxidatively modified in an unfolded state, Ig-domain unfolding occurs already at physiological sarcomere lengths (Rivas-Pardo et al., 2016). Another effect of oxidative stress is a reduced activity of NOS, resulting in lower amounts of NO (De Pascali et al., 2014). Since PKG activity depends on NO availability, oxidative stress can cause hypo-phosphorylation of the N2-B*us* (Beckendorf and Linke, 2015) (Section 5.1).

Within the PEVK domain a ubiquitination site (K11877) is directly neighbouring the well-established PKC*α* phosphorylation site S11878, according to the full-length human titin sequence (UniProtKB accession number, Q8WZ42) (Wagner et al., 2012). Crosstalk between ubiquitination and phosphorylation events have become a regularly discussed topic especially in cell signaling regulation (Hunter, 2007; Nguyen et al., 2013). The PEVK domain has been described as an unstructured, random-coiled region (Watanabe et al., 2002) and, therefore, potential conformational changes caused by incorporation of polyubiquitin chains can hardly be predicted. Nevertheless, it seems likely that posttranslational modifications adjacent to ubiquitination motifs influence not only local signaling events but also ubiquitin-mediated degradation, and vice versa (Section 4.1.1).

## 3 Mechanisms of Protein Quality Control

Intracellular PQC is essential for maintaining the balance between protein degradation and synthesis. In long-lived striated muscle cells, maintaining proteome balance is demanding. A large number of components must be correctly synthesized, folded and incorporated into the sarcomere (Hnia et al., 2019). Imbalance of protein homeostasis leads to accumulation of misfolded proteins and cytosolic aggregation with severe proteotoxicity (Wang and Hill, 2015). Dysregulated PQC has been implicated in various diseases such as cardiovascular diseases (Wang and Hill, 2015; Sciarretta et al., 2018), skeletal muscle myopathies (Kley et al., 2016) and diabetes (Sciarretta et al., 2015). In post-mitotic cardiomyocytes with low regenerative capacity and permanent mechanical stress, it becomes even more complicated as components have to be incorporated into the working sarcomere. Therefore, integration into the Z-disk, the A-band and the M-line or excision of the giant protein titin from the sarcomere to allow further degradation seems to be the *ultimate challenge*. To date, the exact mechanisms of PQC for titin are not fully understood.

**FIGURE 1:**
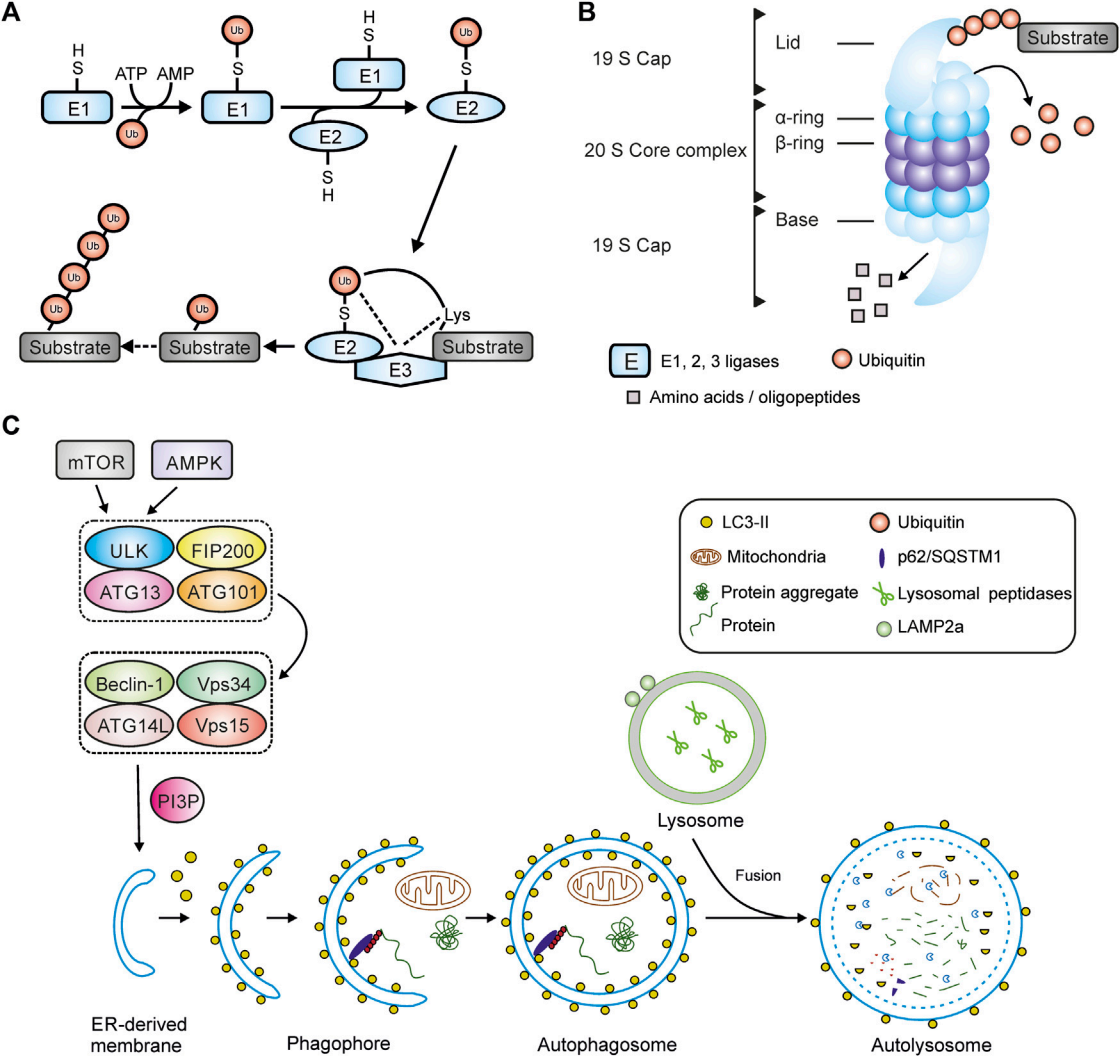
Main components of protein quality control. **(A)** Ubiquitination of a substrate protein by E1-, E2- and E3-ligases. **(B)** Structure of the 26S-proteasome. **(C)** Schematic overview of macroautophagy including autophagy initiation complexes, autophagosome maturation and fusion with a lysosome. Abbreviations: AMPK, AMP activated protein kinase; Atg, autophagy-related (gene); FIP200, focal adhesion kinase family-interacting protein of 200 kD; LAMP2a, lysosomal-associated membrane protein 2a; LC3, microtubule-associated protein 1 light chain 3; mTOR, mammalian target of rapamycin; PI3P, phosphatidylinositol 3-phosphate; ULK1, unc-51–like autophagy-activating kinase 1; Vps34, class III phosphoinositide 3-kinase; Vps15, serine-threonine kinase Vps15/ird1.

**FIGURE 2:**
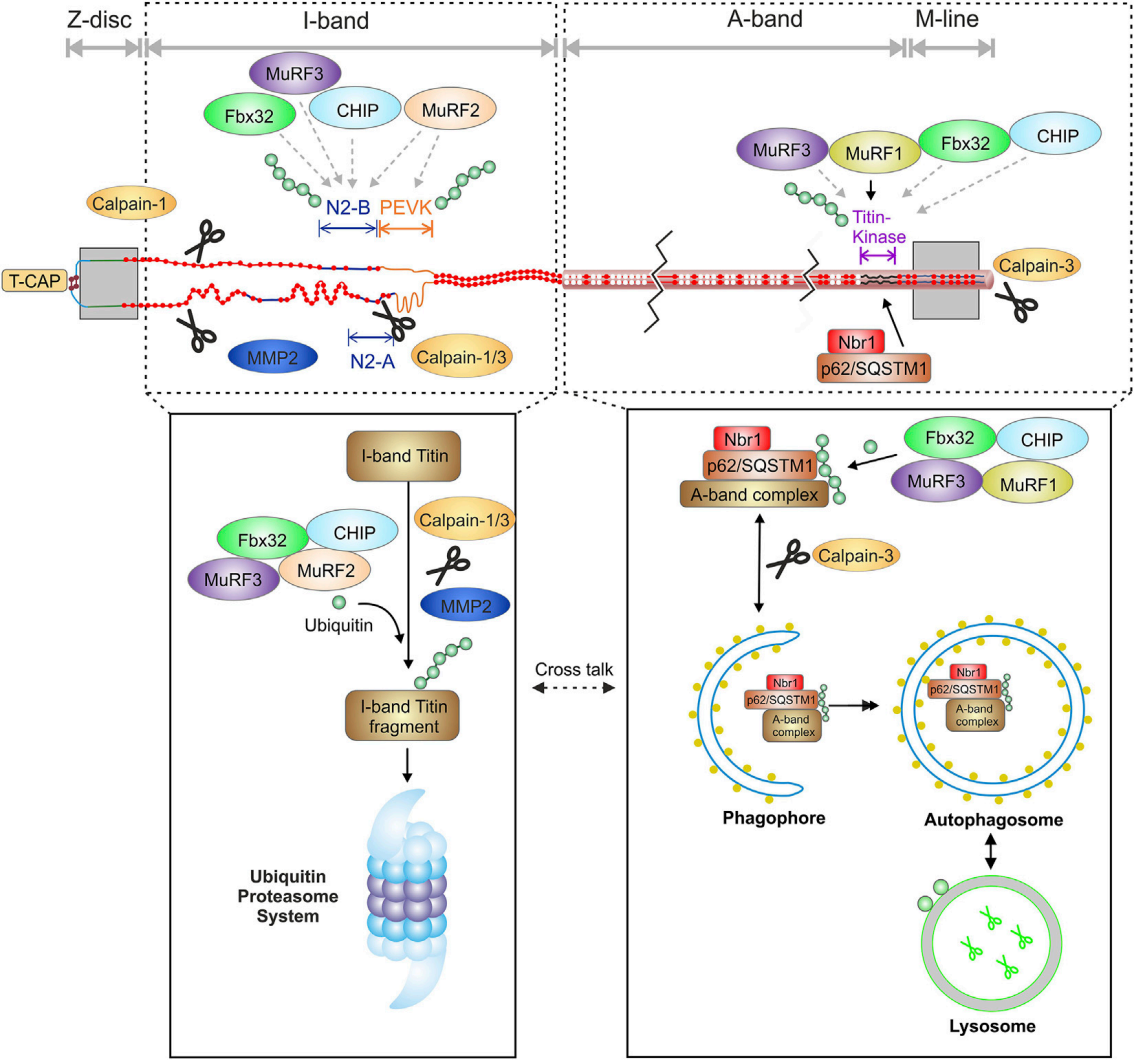
Schematic overview of the interaction of the titin filament with components of the protein quality control and suggested domain specific degradation of titin by the proteasome and autophagy. MMP2, matrix metalloproteinase-2; T-CAP, titin cap/telethonin; N2-B, cardiac specific domain; PEVK, Prolin, glutamate, valine and lysine rich domain.

The following sections provide an overview of the central processes of PQC and summarize the current state of knowledge on the degradation and remodeling of the protein giant titin. Major components of PQC are the ubiquitin-proteasome-system (UPS), the autophagosomal-lysosomal system (autophagy), proteases such as calpains and matrix metalloproteinase 2 and heat shock proteins (Figures 1, 2).

Degradation usually begins with the labeling of target proteins by polyubiquitin chains (Section 3.1), which specifically direct the proteins to the respective degradation machinery of the proteasome (Section 3.2) or autophagosome (Section 3.3). Depending on the substrate, additional pre-digestion by proteases is required to make the substrates accessible for further degradation (Section 3.4). In the sarcomere, calpains have been shown to be involved in the disassembly of myofilament complexes so that they can be degraded by the proteasome (Kramerova et al., 2005; Galvez et al., 2007). Heat shock proteins (HSPs) take on the role of protective chaperones, primarily to prevent misfolding and premature degradation (Section 3.5). In the following section, the individual stations will be discussed in more detail.

### 3.1 E3-Ligases

Ubiquitination of proteins is a three-step reaction performed by E1 (ubiquitin-activating), E2 (ubiquitin-conjugating) or E3 ligases (ubiquitin-ligating) (Figure 1A). Substrate specificity is based on the diversity and large number of E3 ligases performing the final step (Wang et al., 2011). To date, 2 E1-ligases and 38 E2 ligases have been identified within human tissues (Gundogdu and Walden, 2019). In contrast, hundreds of E3-ligases are known that can be classified into different types. The Really Interesting New Gene (RING) family is the largest type of E3 ubiquitin ligases with more than 600 members in humans alone (Morreale and Walden, 2016). Other families are the Homologous to E6AP C-terminus (HECT) E3 ubiquitin ligases (Lorenz, 2018), the Cullin-RING ubiquitin ligases (CRLs) and the U-box containing E3 ligases, also named E4 ligases (Morreale and Walden, 2016) and Ring Between Ring (RBR) E3-ubiquitin ligases (Dove and Klevit, 2017). Several muscle specific E3-ligases have already been identified in the past including the members of the muscle specific ring finger (MuRF) family members MuRF-1, -2, -3, also called tripartite motif containing (TRIM), and F-box protein 32 (Fbx32) also called atrogin-1 or MAFbx (Willis et al., 2008). In cardiomyocytes MuRF-1 negatively regulates cardiac hypertrophy through proteasomal degradation of calcineurin. Mutations in the gene encoding MuRF-1 have been found in patients with cardiac hypertrophy (Chen et al., 2012). Fbx32 is another muscle specific E3-ligase that, like MuRF-1, promotes protein degradation and is thought to inhibit cardiac hypertrophy. In contrast, inhibition of Fbx32 supresses hypertrophy through subsequent activation of nuclear factor kappa B signaling (Usui et al., 2011). MuRF-2 plays an important role in myofibril assembly in neonatal cardiomyocytes (Perera et al., 2011). MuRF-1 and Fbx32 are known to be key regulators of skeletal muscle atrophy and are involved in proteasomal degradation of myosin and myosin binding protein C (Gomes et al., 2001; Clarke et al., 2007; Cohen et al., 2009).

**FIGURE 3:**
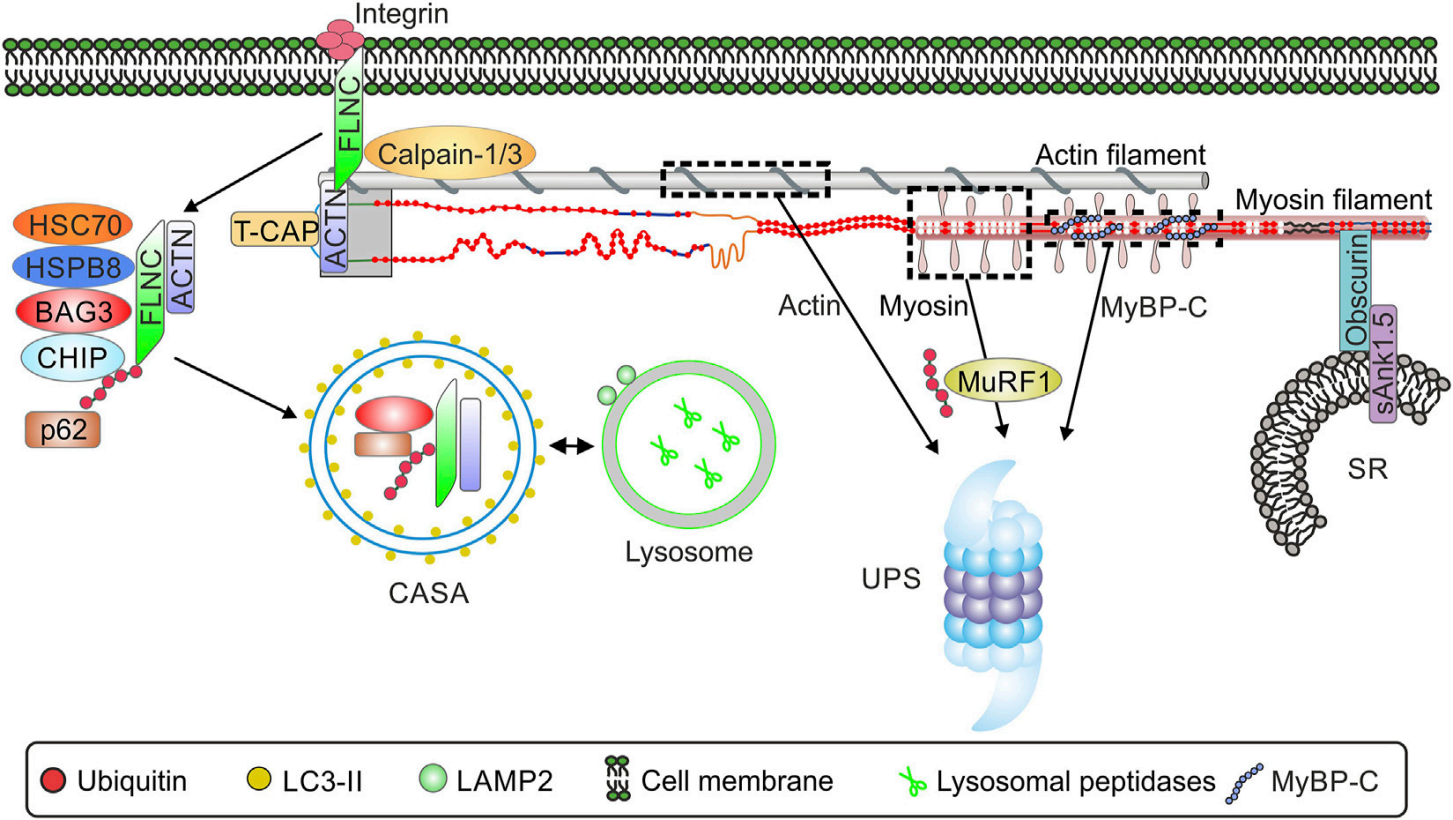
Schematic overview of the interaction of sarcomere and selected sarcomere-associated proteins and protein quality control. Abbreviations: ACTN, *α*-actinin; BAG3, Bcl2-associated anthanogene 3; CASA, chaperone assisted selective autophagy; CHIP, Carboxy terminus of HSP70 interacting protein; HSP70, heat shock protein 70; HSPB8, heat shock protein family B member 8; LAMP2, Lysosome-associated membrane protein 2; LC3, microtubule-associated protein 1 light chain 3; MuRF-1, muscle specific ring finger 1; p62, sequestosome-1/p62; sAnk1.5, Small ankyrin-1 isoform 5; FLNC, filamin-C ; UPS, ubiquitin-proteasome system; SR, sarcoplasmatic reticulum. TCAP, telethonin/titin-cap.

Carboxy terminus of HSP70 interacting protein CHIP (also named STUB1) is expressed in several tissues, but shows highest expression levels in cardiac and skeletal muscle. CHIP is capable of forming both K48 as well as K63 polyubiquitin chains (Zhang et al., 2005; Shaid et al., 2012) suggesting a role in proteasomal and autophagosomal degradation of substrate proteins or organelles. CHIP is critical for quality control processes and ubiquitinates misfolded proteins when correct folding cannot be achieved (Demand et al., 2001). It plays a cardioprotective role during ischemia and reperfusion by increasing PQC in a PKG-dependent manner (Ranek et al., 2020). In a complex with Bcl2-associated anthanogene 3 (BAG3), HSPB8, HSC70 and p62, CHIP is involved in the degradation of Z-disk components including filamin c (Figure 3) (Arndt et al., 2010). It has been reported that BAG3/CHIP dependent sarcomere protein turnover is essential for maintenance of myofilament function (Martin et al., 2021).

In skeletal muscle, MuRF-1 and 2 are reported to ubiquitinate titin fragments from the M-line-A-band transition zone (Witt et al., 2005; Higashikuse et al., 2019). Inhibition of MuRF-1 by small molecules improves diastolic function in heart failure with preserved ejection fraction (HFpEF) and attenuates skeletal muscle wasting in cardiac cachexia (Bowen et al., 2017; Adams et al., 2022). Recently, the involvement of MuRF-1, -2, -3, Fbx32 and CHIP in proteasome- and autophagosome dependent titin filament ubiquitination has been demonstrated (Section 4.1 and Figure 2) (Müller et al., 2021).

### 3.2 Ubiquitin-Proteasome System

The ubiquitin-proteasome system (UPS) is a large, multi-subunit protease complex that functions in an ATP-dependent manner and is responsible for the regulated non-lysosomal degradation of most misfolded or defective cytoplasmic proteins (Livneh et al., 2016). Proteins degraded by the proteasome must be tagged with a poly-ubiquitin chain (Lander et al., 2012), however, mono-ubiquitination of some substrates has also been reported to lead to proteasomal degradation (Braten et al., 2016). Ubiquitin is a small, highly conserved protein composed of 76 amino acids. *Via* the N-terminal methionine, ubiquitin molecules are bound to lysine residues of substrate proteins. Polyubiquitin chains consist of at least four ubiquitin molecules that are linked to each other *via* different lysine residues within the ubiquitin molecule (K6, K11, K27, K29, K33, K48, K63) (Swatek and Komander, 2016). In proteasomal degradation, the linkage occurs *via* K48 (Thrower et al., 2000).

A catalytic 20S core and one or two regulatory 19S subunits form the 26S proteasome (Bard et al., 2018) (Figure 1B). The 20S core is arranged in a stack of four rings, each with 7 subunits, two *α*-rings (*α*1-*α*7) and two *β*-rings (*β*1-*β*7) (Lander et al., 2012). Degradation of substrate proteins occurs through three key protease activities: caspase-like activity (*β*1-subunit), trypsin-like (*β*2-subunit) and chymotrypsin-like activity (*β*5-subunit). These subunits can be replaced by the inducible *β*1i, *β*2i and *β*5i subunits under different physiological and pathophysiological conditions (Scruggs et al., 2011). The 19S regulatory subunit consists of 19 subunits that can be divided into the lid and the base. The base consists of six regulatory particle AAA ATPase subunits (Rpt1-Rpt6) building a ring structure and four non-ATPase subunits (Rpn1, Rpn2, Rpn10 and Rpn13) (Díaz-Villanueva et al., 2015). The lid contains nine different subunits (Rpn3, Rpn5-9, Rpn11, Rpn12 and Rpn15). Ubiquitinated proteins are recognized by the ubiquitin binding proteins Rpn1, 10 and 13 within the 19S lid and ubiquitin chains are partially removed and rescued from degradation primarily by the Rpn11 subunit (Verma et al., 2002; Yao and Cohen, 2002). The substrates are unfolded and transported through a narrow central pore into the catalytic core where they are degraded (Lander et al., 2012). This central pore is formed by the N-termini of a subgroup of *α*-subunits that blocks the unregulated entry of substrates into the catalytic core (Groll et al., 2000).

### 3.3 Autophagy

The autophagosomal-lysosomal system processes individual proteins, larger protein complexes, aggregates and complete organelles (Shaid et al., 2012). It is regulated by specific autophagy-related genes (Atg) that control the initiation, maturation and fusion of autophagosomes with lysosomes to form autolysosomes (Sciarretta et al., 2018). To date, more than 35 Atg genes have been identified, most of which are conserved between yeast and mammals (Ravikumar et al., 2010). Autophagy is activated by various stresses such as nutrient/glucose deprivation or oxidative stress (Sciarretta et al., 2018). In recent years, autophagy has gained attention as an important regulator of cardiac protein homeostasis and function. By eliminating misfolded proteins and damaged organelles, autophagy is central to maintaining cardiac structure and function (Nakai et al., 2007; Ikeda et al., 2015). There are three different types of autophagy: macroautophagy, chaperone-mediated autophagy (CMA or CASA for chaperone assisted selective autophagy) and microautophagy. In CMA, soluble substrates containing a KFERQ sequence are bound to the chaperone heat shock cognate protein 70 (HSC70/HSPA8), and the proteins are delivered directly to the lysosome by binding to lysosome-associated membrane protein 2a (LAMP2a) (Arndt et al., 2010). In the process of microautophagy, a small portion of the cytosol is directly engulfed by the lysosomal membrane. Macroautophagy describes the degradation process of proteins, protein aggregates, macromolecules or cellular organelles, which involves encapsulation in a double-membraned vesicle, the autophagosome (Shaid et al., 2012). In a first step, small endoplasmatic/sarcoplasmatic reticulum-derived vesicular sacs form around the target proteins, the autophagophore. Substrates are anchored to the membrane by autophagy-related proteins such as microtubule-associated light chain 3 (LC3/Atg8) and the ubiquitin binding protein p62/SQSTM1 (Shaid et al., 2012). Once the membrane closes around the target substrates, it is called autophagosome. Eventually, the autophagosome is fused to a lysosome and the substrates are degraded by lysosomal proteases (Levine and Kroemer, 2008; Gatica et al., 2015). In the following macroautophagy is referred to as autophagy. For autolysosomal degradation, unfolded protein substrates and protein aggregates are also labelled by polyubiquitin chains. In this case the ubiquitin molecules are typically linked *via* lysine residue 63 (K63) (Ji and Kwon, 2017).

A key element of autophagy initiation is the autophagy-initiating kinase Ulk1 (mammalian homologue of yeast ATG1) as part of the ULK1-ATG13-FIP200 protein complex (Mizushima, 2010). ULK1 contains multiple phosphorylation sites that regulate their activity when targeted e.g., by AMP activated protein kinase (AMPK) or mammalian target of rapamycin (mTOR) (Kim et al., 2011). Under glucose starvation, AMPK-mediated phosphorylation of ULK1 (e.g., at S317, S777 and S555) activates autophagy (Egan et al., 2011; Kim et al., 2011). In contrast, mTOR, a cell growth regulator, associates with the ULK1-ATG13-FIP200 complex, leading to phosphorylation of ULK1 at S757, thereby disrupting the AMPK-ULK1 interaction and inhibiting autophagy (Egan et al., 2011). Activated ULK1 phosphorylates and recruits a class III phosphoinositide-3-kinase (PI3-K) complex to the site of formation of the isolation membrane to start phagophore assembly (Feng et al., 2014) (Figure 1C).

### 3.4 Calpains and MMP2

The calpain family in humans consists of 15 members and all are Ca^2+^-dependent, non-lysosomal cysteine proteases (Chen et al., 2021). Unlike other proteases that catalyse the degradation of their substrates, calpains recognize and proteolyze their substrates, but do not degrade them (Ono and Sorimachi, 2012). Three members have been identified in muscle tissues, calpain-1 (µ-calpain) and -2 (m-calpain) are ubiquitously expressed whereas calpain-3 is predominantly found in skeletal muscle (Portbury et al., 2011; Letavernier et al., 2012). The inhibitory protein calpastatin serves as the primary negative allosteric modifier of calpain activity by interaction between calpain and inhibitory domains of calpastatin (Hyatt and Powers, 2020). All three calpains have been associated with the degradation of sarcomere proteins including titin (Lim et al., 2004; Portbury et al., 2011).

Matrix metalloproteinase 2 (MMP2) is a zinc-dependent protease that degrades components of the extracellular matrix. Although normally localized in the extracellular matrix, MMP2 does not exclusively degrade matrix components and intracellular and particularly sarcomeric localization of MMPs has been demonstrated (Ali et al., 2010). Under increased oxidative stress, MMP-2 has been shown to degrade various sarcomere proteins such as troponin, myosin-light chain and *α* -actinin (Wang et al., 2002; Gao et al., 2003; Sawicki et al., 2005; Sung et al., 2007). In cardiomyocytes intracellular MMP2 can be found in the Z-disc region where it has been suggested to degrade the titin filament, particularly after experimental ischemia/reperfusion. (Ali et al., 2010).

### 3.5 Heat Shock Proteins

Another important element of PQC are the heat shock proteins that help other proteins fold or maintain their secondary structure under extreme conditions. They are produced in increased amounts after cells have been exposed to heat or other types of stressful environmental influences such as ultraviolet radiation, heavy metals or ethanol. In these situations of cellular stress, heat shock proteins stabilise cellular proteins to protect them from denaturation or accelerate the degradation of non-functional proteins *via* the proteasome. There are several different types of heat shock proteins, including the ATP-dependent HSP90 and HSP70, chaperonin containing TCP1 [CCT; also called TCP1-ring complex (TRiC)] and the non-ATP-dependent small heat shock proteins (sHSP) (Willis and Patterson, 2010). sHSPs, a family of molecular chaperones with 10 members in humans (HSPB1-10) (Kappé et al., 2010) and a molecular weight of 12–43 kDa, play a central role the PQC machinery. Despite their low molecular weight, sHSPs can form huge oligomers of up to several hundred kilo Dalton (Vos et al., 2008). As non ATP-dependent chaperones, sHSPs are not able to refold, but bind damaged or partially unfolded client proteins to keep them in a folding-prone state and protect them from aggregation (Mymrikov et al., 2011). Under several stress conditions like oxidative stress or energy depletion expression levels of sHSPs are increased in order to protect the cell (Mymrikov et al., 2011). Overexpression of HSP20 (HSPB6) protects against ischemia/reperfusion injury by activating autophagy (Fan et al., 2005; Qian et al., 2009). HSP22 (HSPB8) and HSP27 (HSPB1) have also been shown to have a protective effect after ischemia/reperfusion (Vander Heide, 2002; Depre et al., 2006). HSP27 and *α*B-crystallin (HSPB5) are ubiquitously expressed in mammalian tissues (Klemenz et al., 1993). The chaperone activity of HSP27 and *α*B-crystallin is regulated by their oligomeric structure and the phosphorylation status (Ahmad et al., 2008; Jovcevski et al., 2015). *α*B-crystallin interacts with the 20S core of the proteasome and phosphorylation-independent with Fbx4, a subunit of the ubiquitin ligase complex SCF, suggesting the degradation of bound substrates by the proteasome (Boelens et al., 2001; den Engelsman et al., 2003). HSP27 has been shown to interact with the 19S unit of the proteasome and with ubiquitin, suggesting a role in substrate degradation by the proteasome also for this heat shock protein (Garrido et al., 2006). Both, HSP27 and *α*B-crystallin can translocate from the cytosol to the sarcomere under different stimuli and pathological conditions (Kötter et al., 2014; Unger et al., 2017).

The ATP-dependent chaperone HSP90 is ubiquitously expressed and has been reported to be a chaperone for myosin in the sarcomere (Smith et al., 2014). HSP90 does not directly interact with titin, but it can associate to the N2A domain of titin in a complex with the titin binding partner SET and MYND domain containing protein 2 (SMYD2) thereby exerting a protective effect on the Z-disc/I-band structure. For this interaction, HSP90 has to be methylated by SMYD2 at lysine K616 (Donlin et al., 2012). Following glutathionylation or oxidation of SMYD2, the complex dissociates and titin/N2A can be degraded by calpain-1 and/or MMP2 pointing to a positive role of SMYD2 and HSP90 in regulation of titin stability (Donlin et al., 2012; Munkanatta Godage et al., 2018).

## 4 Protein Quality Control of Sarcomere Proteins

### 4.1 Protection and Degradation of Titin

As mentioned earlier, controlling the protein quality of the titin filament is particularly challenging because the filament consists of a single protein and replacing a defective titin molecule in a functioning sarcomere is a difficult task. The reported half-life of a titin molecule under baseline conditions is about 3–5 days (Isaacs et al., 1989), but exchange of titin filaments in the sarcomere can occur within hours (da Silva Lopes et al., 2011). During neonatal development titin isoforms switching from foetal isoforms to the adult isoforms occurs within in days in small mammals (Opitz et al., 2004; Krüger et al., 2008) and weeks to months in larger animals (Lahmers et al., 2004). In adult heart, interventions such as pharmacologically induced hypothyroidism (Wu et al., 2007) and transverse aortic constriction (Han et al., 2020) have been reported to alter titin isoform composition within weeks. This process includes incorporation of newly synthesised titin filaments into growing sarcomeres as well as turnover of the foetal isoforms.

It was shown that translation of titin mRNA occurs predominantly at the Z-disk and the I-band of the sarcomere, i.e., in close proximity to the subsequent site of incorporation (Rudolph et al., 2019). The study by Rudolph et al. also partially refutes the long-discussed hypothesis of gradual integration of the titin filament into the Z-disc and subsequently into the M-lineage, by showing the placement of fluorescently labelled full-length titin filaments in the sarcomere. According to the study, integrated titin filaments originate primarily from a soluble cytosolic pool that represents approximately 10–15% of total titin (Rudolph et al., 2019). The exact mechanisms of titin filament integration into the sarcomere are not resolved yet, however, it is likely impeded by ongoing contraction of the sarcomeres.

Similarly, only little information is available on the mechanisms involved in titin PQC and turnover. Its gigantic size makes titin highly susceptible to fragmentation and degradation, suggesting that a continuous, well-functioning mechanism for protein turnover is required.

The sHSPs *α*B-crystallin and HSP27 as well as HSP90 have been identified as direct or indirect binding partners of titin I-band domains and likely play an important role in the PQC of the giant molecule (Bullard et al., 2004; Kötter et al., 2014). Under ischemic conditions and mechanical stretch of cardiac myocytes, conditions that occur during high mechanical stress, titin domain unfolding and subsequent interfilament aggregation can occur, leading to increased passive stiffness of muscle cells. It could be shown *in vitro*, that the presence of the small heat shock proteins HSP27 or *α*B-crystallin titin aggregation is blocked and an increase in passive stiffness is prevented (Kötter et al., 2014). HSP27 and *α*B-crystallin also showed translocation from the Z-disk to I-band domains e.g., in failing hearts and skeletal muscle dystrophies indicating a protective effect on these domains under pathological conditions. HSP27 and *α*B-crystallin have been shown to interact with the proximal Ig-region, the N2B and the N2A domain but not the PEVK, indicating a translocation to and a protection for these domains (Bullard et al., 2004; Kötter et al., 2014). *In vitro* experiments in human cardiomyocytes from failing hearts showed that treatment with *α*B-crystallin can decrease pathologically increased titin-based passive stiffness (Franssen et al., 2017). Thus, one might speculate that *α*B-crystallin not only prevents the formation of titin aggregates, but can also dissolve them. which would be a finding with significant potential for novel therapeutic strategies. However, caution is required here, because there is no reported evidence at the molecular level that sHSPs alone are capable of refolding proteins or even dissolving aggregates.

Due to the size of a titin filament, its degradation is an extremely complex and probably energy-consuming task for the cell. A first sign of elevated titin degradation is a higher abundance of the T2 fragment with a size of approximately 2–2.5 MDa, a specific degradation intermediate mainly existing of the A-band part of titin (Linke and Hamdani, 2014). The recently identified titin cronos isoform (∼2 MDa) (Zou et al., 2015; Zaunbrecher et al., 2019) appears in the same molecular weight range than the T2 band and cannot be easily distinguished by electrophoresis alone. Gelelectrophoretic separation of full-length titin and the T2 revealed 2, 3 or more separate T2 bands (Neagoe, 2002; Lahmers et al., 2004; Kruger and Linke, 2006; Krüger et al., 2008). Since the A-band portion of titin is identical in most isoforms, the different molecular weights of the T2 product indicate a different composition of the I-band domain of the degradation intermediate, similar to variable I-band length in the N2BA isoforms. This could also imply that multiple excision sites exist within the titin I-band resulting in different degradation products.

Current evidence suggests that both the proteasome (Kötter et al., 2016; Higashikuse et al., 2019) and autophagy (Lange et al., 2005; Bogomolovas et al., 2021; Müller et al., 2021), are involved in the degradation of titin and that the degradation of the different titin domains might occur specifically *via* one or the other system. This hypothesis was lately supported by experimental *in vitro* inhibition of the proteasome, which lead to increased proteasome-dependent K48 polyubiquitination, and inhibition of autophagy, causing increased autophagy-dependent K63 polyubiquitination of titin (Kötter et al., 2016; Müller et al., 2021).

The observed titin ubiquitination is at least in part performed by the muscle specific E3-ligases MuRF-1, -2, -3, CHIP and Fbx32/atrogin-1. Importantly, polyubiquitination occurs at full-length titin indicating is fully accessible for ubiquitination while still incorporated into the sarcomere (Müller et al., 2021). This is in accordance with other studies showing ubiquitination of other sarcomere proteins within the sarcomere (Martin et al., 2021). It can therefore be concluded that also for a giant protein like titin, a predigest is not necessarily needed for the ubiquitination reaction itself (Müller et al., 2021). Nevertheless, predigest of titin is important for further processing by autophagy or the proteasome and could be performed by the proteases calpain 1 and/or 3 as well as MMP2 (Barta et al., 2005; Hayashi et al., 2008; Azevedo et al., 2014). Calpain-1 has been identified as a binding partner of the N2A domain and the proximal Ig-domains of titin (Raynaud et al., 2005; Coulis et al., 2008). *In vitro* experiments have demonstrated that calpain-1 proteolyzes recombinant titin N2A constructs, suggesting that N2A is one of the titin subdomains susceptible to proteolysis by calpain-1 (Hayashi et al., 2008). The sequences within N2A that are degraded by calpain-1 were analyzed and mapped to two sites at the N- and C-terminal borders of N2A’s unique insertion (uN2A). Calpain-3 also interacts with the N2A domain of titin (Hayashi et al., 2008). The release of calpain-3 from the N2A domain seems to be necessary for the activation and proteolysis of titin (Adewale and Ahn, 2021). Calpain-3 additionally binds to the C-terminal part of titin in the M-line region and leads to its proteolysis (Sorimachi and Ono, 2012; Charton et al., 2015).

MMP2 has been shown to associate to titin in Z-disk proximity at regions that share >60% sequence identity with MMP2 cleavage sites (Ali et al., 2010). The Z-disk proximity of MMP2 binding makes it highly likely that MMP2 is preferentially involved in the degradation of the readily accessible I-band region of titin. Calpains and MMP2 digest I-band titin into smaller fragments that can be readily recognized and degraded by the proteasome. This supports the idea that the accessible I-band part of titin could be preferentially degraded *via* the proteasome. Of note, the specific titin degradation band T2 was increased after inhibition of autophagy or the proteasome suggesting that under these conditions the titin degradation is stalled after proteolytic pre-digest, resulting in accumulation of the pre-digest product T2 containing titins A-band (Müller et al., 2021). The A-band portion of titin, which is tightly linked to myosin and myosin-binding protein C (Linke and Hamdani, 2014) is thought to be embedded in an autophagosome and degraded after fusion with a lysosome. The hypothesis of domain dependent degradation is supported by recent studies demonstrating localization of proteasome subunits of the 20S core at the Z-disk/I-band region of the sarcomere in adult cardiomyocytes (Rudolph et al., 2019) and localization of the autophagy-related Nbr1/p62/MuRF-1 complex at the titin kinase region upon MuRF-1 mediated ubiquitination of titin (Lange et al., 2005; Bogomolovas et al., 2021). Figure 2 summarizes recent findings supporting the hypothesis of a domain specific degradation of titin by the proteasome and autophagy.

#### 4.1.1 Potential Degradation-independent Effects of Titin Ubiquitination

Given the important role of titin as a mechanosensitive protein and its function as a molecular spring excessive modification of the protein may also have significant effects on its biomechanical properties. A polyubiquitin chain of 5 molecules has a molecular weight of about 40 kDa, so the size in itself could lead to steric hindrance of other posttranslational modifications. Whether titin ubiquitination directly or indirectly affects titin mechanical properties remains to be investigated. In mice, a previous study reported ubiquitination of titin within the PEVK domain at lysine residue K11877 (Wagner et al., 2012). This site is adjacent to a well-described PKC*α* phosphorylation site (S11878) that regulates titin-based passive tension. It can be speculated that mono- and/or poly-ubiquitination of K11877 alters the accessibility of the phosphorylation site, thereby preventing phosphorylation or de-phosphorylation of the S11878 residue. Such changes in the phosphorylation status of titin may in turn affect its elastic properties and cardiomyocyte function. Monoubiquitination of titin, e.g., within the N2-B domain, may also have implications for titin-based signal transduction by altering hypertrophic signaling *via* the N2B/four and a half LIM domain protein 1 and 2 (FHL1, FHL2)/MAPK complex.

### 4.2 Degradation of Other Sarcomere Proteins

Several studies have shown that the E3 ligase MuRF-1 plays a key role in the degradation of sarcomere proteins. MuRF-1 ubiquitinates myofibrillar proteins such as troponin I (Kedar et al., 2004), myosin heavy chains (Clarke et al., 2007; Fielitz et al., 2007), actin (Polge et al., 2011), myosin binding protein C and myosin light chain 1 and 2 (Cohen et al., 2009). Subsequently, all of these substrates are degraded by the proteasome (Figure 3).

The giant protein obscurin, binding partner of titin and myomesin (Fukuzawa et al., 2008), is localized in the M-band of the sarcomere. Small ankyrin-1 isoform 5 (sAnk1.5) is localized at the membrane of the sarcoplasmatic reticulum (SR) from where it interacts with the C-terminus of obscurin in cardiac and skeletal muscle (Lange et al., 2009) (Figure 3). This structural interaction of sarcomeric obscurin and sAnk1.5 in the membrane of the SR is necessary to anchor the SR in close proximity to the myofibrils. In the absence of sAnk1.5 longitudinal SR architecture is disrupted (Giacomello et al., 2015). In addition, interaction with obscurin prevents the degradation of sAnk1.5. Disruption of thi obscurin-sAnk1.5 interaction leads to dislocation of sAnk1.5 to the sarcomeric Z-disc and cullin-3-mediated degradation (Lange et al., 2012).

The chaperone UNC-45 promotes folding and formation of myosin molecules (Gaiser et al., 2011; Melkani et al., 2011). Together with HSP70 and HSP90, UNC-45 builds a multisite docking platform for the assembly of myosin filaments (Gazda et al., 2013). Mutations in conserved regions of UNC-45 leading to reduced protein levels and/or chaperone activity results in paralysis and dysregulated thick filaments in *Caenorhabditis elegans* (Moncrief et al., 2021).

Another important mediator of sarcomere protein degradation is the co-chaperone BAG3 (Ulbricht et al., 2013; Behl, 2016). In skeletal muscle, BAG3 induces the formation of a protein complex that includes HSC70, the small heat shock protein HSP22 (HSPB8) and the carboxy-terminus of HSP70 interacting protein CHIP (Arndt et al., 2010). CHIP ubiquitinates the chaperone-bound substrate proteins and BAG3, facilitating interaction of the complex with p62 and potentially resulting in co-degradation of the ubiquitinated substrate and BAG3. The complex induces the degradation of Z-disk components, such as filamin-C by chaperone assisted selective autophagy (CASA, Figure 3) (Arndt et al., 2010). The exact function of filamin-C has not yet been elucidated. Filamin-C is found in subsarcolemmal regions as well as at Z-disks, so it is likely that the function of filamin-C is to act as a communication pathway between the membrane and the sarcomere. Alterations in the subcellular localization of filamin-C have been noted in limb-girdle muscular dystrophy and Duchenne muscular dystrophy (Mao and Nakamura, 2020). Filamin C is necessary for muscle formation and in the absence of FLNC mice develop a severe muscle phenotype including defects in embryonic myogenesis with decreased number of primary fibers, massive fiber size variation, and a disturbed sarcomere architecture (Dalkilic et al., 2006). The importance of the BAG3-dependent CASA mechanism has also been demonstrated in cardiac muscle, for which 8 proteins have been identified to date whose degradation is dependent on BAG3, including the sarcomere proteins *α*-actinin and the myosin-binding protein C. Insufficient turnover of sarcomere proteins as a result of impaired BAG3/CHIP-mediated degradation has been suggested to lead to impaired contractility of the myocardium (Martin et al., 2021).

## 5 Pathological Changes of Protein Quality Control in Heart and Skeletal Muscle

Striated muscle is a tissue with very high metabolic activity and is subject to strong mechanical stress. For this reason, highly effective and precisely regulated protein homeostasis is critical for cardiac and skeletal muscle function. As described in the previous sections, the UPS and autophagy play a central role in protein homeostasis. While the proteasome is already constantly active under normal conditions, autophagic activity is usually low in the heart (Levine and Kroemer, 2008). Dysregulation of proteasome and autophagosomal activity occurs in response to stress situations and diseases like ischemia/reperfusion, cardiac hypertrophy, heart failure and skeletal muscle atrophy (Gustafsson and Gottlieb, 2008; Portbury et al., 2011; Schlossarek and Carrier, 2011; Schiaffino et al., 2013) In addition, metabolic diseases such as diabetes are associated to alterations in proteasomal and autophagosomal activity (Queisser et al., 2010; Xu et al., 2013). The accumulation of ubiquitinated proteins may indicate impaired or overloaded UPS and/or autophagy, and is commonly observed in various muscle diseases. Accumulation of misfolded protein is an age-related symptom in mammals caused, at least partially, by downregulation of the activity of the UPS (Tomaru et al., 2012) and the autophagosomal system (Shirakabe et al., 2016). Aging affects several cellular targets. Titin isoform composition or titin based passive stiffness do not seem to be altered during aging but proteasomal activity and calpain-1 activity declines during aging leading to an accumulation of aggregated proteins (Salcan et al., 2020). According to various studies, autophagic activity also appears to generally decrease during aging (Aman et al., 2021) Only for aged primary human fibroblast cells an increased autophagic activity for the turnover of ubiquitinated proteins has been reported (Gamerdinger et al., 2009). For this reason, it has to be considered that in some pathological situations, especially in heart diseases, PQC could also be affected by aging alone. The following sections will briefly review a selection of pathological changes observed in the context of striated muscle diseases, again with a specific focus on their impact on protein homeostasis and function of the sarcomere protein titin.

### 5.1 Altered Protein Quality Control in Response to Cardiac Ischemic Injury

Myocardial ischemia is characterized by a brief interruption of blood supply to the working myocardium, resulting in a greater or lesser degree of myocardial tissue death depending on the duration of ischemia. Additional tissue damage occurs during the reperfusion phase and is a result of complex processes such as increased production of reactive oxygen species (ROS), impaired Ca^2+^ handling, and altered cellular metabolism (Kalogeris et al., 2012). During ischemia/reperfusion the UPS becomes dysfunctional by oxidative modification of 20S core subunits and loses the ability to process ubiquitinated substrate proteins (Bulteau et al., 2001; Powell et al., 2005). In contrast, autophagosomal activity is increased during ischemia/reperfusion by activation of the AMPK pathway and inhibition of the Rheb/mTOR pathway (Matsui et al., 2007; Sciarretta et al., 2012), apparently to compensate for the greater amount of damaged and ubiquitinated proteins due to an impaired or overloaded UPS. In turn, experimental activation of autophagy during ischemia has been shown to be cardioprotective and to reduce infarct size (Mizushima and Komatsu, 2011; Choi et al., 2013; Kroemer, 2015; Mo et al., 2016). However, during reperfusion, the massive activation of autophagy through beclin-1-dependent but AMPK-independent pathways could be maladaptive (Shi et al., 2019). One explanation for the deleterious effect of hyperactivated autophagy could be the induction of autosis leading to death of cardiomyocytes and further contractile impairment of the affected ventricle (Liu et al., 2013).

Oxidative stress plays an important role in cardiac damage of infarcted hearts and is known to induce modifications of sarcomeric and Ca^2+^-handling proteins, thereby altering their function, expression and activity. This oxidative stress can be mediated by increased production of ROS, such as superoxide radical (O2^•-^) hydroxyl radical (OH^•-^) and the non-radical reactive species hydrogen peroxide (H_2_O_2_).

It is suggested that oxidative stress may be increased beyond physiological levels not only in the infarcted but also in the non-ischemic regions of the heart (remote myocardium) after myocardial infarction (Cave et al., 2006; Sun, 2009). Oxidative stress strongly affects myofibrillar proteins, e.g., by increasing proteolysis of myofilament proteins by ROS-activated proteases (Steinberg, 2013). Oxidative changes in actin and myosin have also been observed during reperfusion of ischemic rat hearts (Eaton et al., 2002; Prochniewicz et al., 2008). Myosin Ca^2+^-ATPase activity was decreased after experimental induction of myocardial infarction (MI) (Avner et al., 2012). Moreover, glutathionylation levels in the myofibrillar protein fraction of the remote myocardium were greatly increased after infarction.

*In vitro* analyses have shown that S-glutathionylation and disulfide bonding of titin fragments could alter the elastic properties of titin in response to oxidative stress (Grützner et al., 2009; Alegre-Cebollada et al., 2014). The authors demonstrated that under conditions of high mechanical strain titin Ig-domains are unfolded and become S-glutathionylated in the presence of oxidized glutathione (GSSG), which then prevents the re-folding of the domain and lowers titin-based stiffness. However, after myocardial infarction S-glutathionylation of unfolded I-band Ig-domains of titin was increased only in the infarcted area, but not in the remote region. (Loescher et al., 2020).

During ischemia-induced sarcomere breakdown titin is among the first proteins to be degraded (Hein et al., 1994). In response to ischemic injury, MMP2 translocates to the Z-disk/I-band region and contributes to titin degradation (Ali et al., 2010). Cardiac Ischemia is associated with both hypoxia and acidosis due to increased glycolysis and lactic acid production of the ischemic myocytes (Webster et al., 2000). The resulting acidic conditions in combination with the increased mechanical load of the sarcomeres are likely to induce Ig-domain unfolding and aggregation of titin filaments as recently demonstrated *in vitro* (Kötter et al., 2014). Such aggregation leads to increased titin-based passive tension. This is in partial conflict with the reported elevated S-glutathionylation of unfolded Ig-domains having the opposite effect (Loescher et al., 2020). Nevertheless, the presence of *α*B-crystallin or HSP27 could prevent aggregation of unfolded titin domains and the increase in passive stiffness in this setting.

Titin phosphorylation and titin-based stiffness was also increased within the viable remote myocardium of mice 3 days after permanent ligature of the LAD. This is thought to be a protective mechanism to stabilize and promote the function of the vital myocardium after massive loss of cardiomyocytes in the infarcted region. As long as the scar within the infarct region is not formed, rapidly increased titin stiffness in the non-ischemic region may prevent load-induced overstretching and disruption of non-ischemic cardiac myocytes and thereby reduce sarcomeric and cellular damage in the first hours and days after myocardial injury (Kötter et al., 2016). It cannot be excluded that aggregation contributes to the observed increase in passive stiffness and the stability of the remote myocardium. While titin aggregation could possibly help stabilizing the sarcomere under conditions of high mechanical stress, aggregation cannot be easily reversed to retrieve functional filaments, and aggregated titin filaments eventually lose their elastic properties. In addition, titin aggregation has been reported to occur in the area of the N2A domain, a region of calpain-1 interaction in heart muscle (Barta et al., 2005). This interaction is potentially affected by aggregation leading to higher calpain activity and proteolysis of titin and other substrates. The increased mechanical stress on the remaining vital myocardium results in greatly increased proteasomal activity. However, despite the higher proteasomal activity in remote myocardium, erosion and ubiquitination of titin appeared to be too high to be compensated by increased proteasomal turnover, and thus led to the accumulation of K48-dependent poly-ubiquitinated titin (Kötter et al., 2016). In the end partially damaged and ubiquitinated titin filaments may remain within the sarcomere and could negatively affect cardiomyocyte function.

Taken together, the exact interplay of the described mechanisms and their role in the functional adaptation of sarcomere function after myocardial infarction or ischemia/reperfusion injury requires further investigation.

### 5.2 Muscle Atrophy and Skeletal Muscle Myopathies

Muscular atrophy occurs when the rate of muscle protein breakdown/turnover exceeds muscle protein synthesis. It is a predominant symptom of several pathologies including muscle myopathies or cardiac and cancer cachexia (Bonaldo and Sandri, 2013). A hallmark of muscle atrophy is a massive loss of skeletal muscle triggered by activation of the UPS and autophagy (Schiaffino et al., 2013). High muscle loss apparently includes increased sarcomere protein turnover.

Lang et al. reported changes in titin ubiquitination, particularly within the C-terminal region, after sciatic nerve section in mouse gastrocnemius muscle, indicating elevated titin turnover during atrophy (Lang et al., 2017). The E3-ligases MuRF-1, that targets several sarcomeric proteins like myosin or MyBP-C for degradation by the UPS (Section 4.2), and Fbx32/atrogin-1 are strongly upregulated during muscle atrophy (Bodine et al., 2001; Gomes et al., 2001). Since titin has been shown to be a polyubiquitination target of both these E3-ligases, significant degradation of titin during atrophy/cachexia development is likely (Müller et al., 2021).

Hereditary myopathies are a heterogeneous group of disorders with a highly variable age at onset. These myopathies are due to mutations in several genes, including dystrophin (Duchenne muscular dystrophy, DMD), calpain-3 (Limb girdle muscular dystrophies, LGMD2A), or titin [muscular dystrophy with myositis (MDM); tibial muscular dystrophy (TMD) and limb-girdle muscular dystrophy 2J (LGMD2J)] (Hackman et al., 2002; Huebsch et al., 2005; Udd et al., 2005). DMD is an X-linked inherited muscular dystrophy based on mutations in the dystrophin gene and is the most common form of dystrophy in childhood (Koenig et al., 1987). Typically, the muscles of the thighs and pelvis affected first. In later stages of the disease, the heart is also concerned, leading to the development of cardiomyopathy (Ryder et al., 2017). Autophagic activity is decreased in DMD patients and in mdx mice by constitutive activation of mTOR, which leads to downregulation of Atg proteins and inhibition of autophagy function (De Palma et al., 2012). In the urine of DMD patients and in mdx mice N-terminal titin fragments have been identified, which may have the potential to serve as non-invasive biomarkers of DMD (Robertson et al., 2017).

LGMD2A is an autosomal recessive human muscular dystrophy characterized by muscle wasting, cell death and decreased calpain-3 activity and expression (Nishino and Ozawa, 2002). MDM is caused by a deletion mutation of 83 amino acids that includes part of the titin N2A domain responsible for the interaction with calpain-3 (Huebsch et al., 2005; Nishikawa et al., 2020). Deletion of this fragment in skeletal muscle of mdm mice results in reduced association of calpain-3 to titin N2A (Huebsch et al., 2005). The lack of interaction between calpain-3 and N2A leads to increased activity of calpain-3 and increased substrate digestion (Huebsch et al., 2005; Nishikawa et al., 2020). Patients with missense or truncating mutation in either of the two last exons of TTN, Mex5 and Mex6, encoding the is7 region and the M10 domain in the M-band proximity, respectively, develop TMD and LGMD2J (Hackman et al., 2008; Evilä et al., 2014).

Myofibrillar myopathies (MFMs) are characterized by histological alterations such as focal disintegration of myofibrils predominantly at the Z-disk and protein aggregation in myofibers (Kley et al., 2016). Overexpression of HSPs significantly reduces aberrant protein aggregation in various models of MFMs (Chávez Zobel et al., 2003; Sanbe et al., 2007; Sanbe et al., 2009). A common feature in hereditary myopathies is the translocation of the small heat shock proteins HSP27 and *α*B-crystallin and the ATP-dependent chaperone HSP90 from the z-disk region or the cytosol to the elastic titin I-band increasing the passive tension of skeletal muscle fibers (Unger et al., 2017). This suggests a protective mechanism that prevents aggregation of unfolded titin domains as well as other affected sarcomeric proteins.

Hereditary myopathy with early respiratory failure (HMERF, MFM-Titinopathy) is a slowly progressive myopathy with onset in adulthood. In addition to the limb girdle and leg muscles, it typically affects the diaphragm muscle, resulting in a severe reduction in forced vital capacity. However, cardiac involvement in the disease has not yet been observed to date (Tasca and Udd, 2018). All HMERF-associated pathogenic titin variants are located in the 119th FN3 domain of titin resulting in misfolding of the FN3 domain *in vitro* (Hedberg et al., 2014; Pfeffer et al., 2014; Tasca and Udd, 2018). In contrast to disease-causing proteins in many other myopathies, the mutant titin protein could not be found in cytoplasmic bodies but in other myofibrillar proteins (Palmio et al., 2014). One possible explanation is the loss of dynamic interaction of the FN3 119-domain with neighbouring proteins, which alters autophagy processes and subsequently leads to aggregation of other proteins (Edström et al., 1990; Tasca and Udd, 2018).

### 5.3 Protein Quality Control in Diabetes Mellitus

Another condition associated with altered PQC is diabetes mellitus (DM). DM describes metabolic diseases that lead to elevated blood glucose levels due to a lack of insulin or a reduced effectiveness of insulin. The pathology of diabetes mellitus is associated with increased morbidity and mortality due to coronary artery disease, peripheral artery disease, stroke, cardiomyopathy and congestive heart failure (King et al., 1998; Plutzky, 2011). Diabetes patients have a 2 fold higher case fatality rate during acute phase of myocardial infarction and long term follow up (Milazzo et al., 2021). Patients with type 2 diabetes mellitus (T2DM) often show a very early manifestation of left ventricular dysfunction due to increased diastolic passive stiffness of the left ventricle. This increased passive ventricular stiffness is associated with cellular and systemic abnormalities often leading to the progression of heart failure (Boyer et al., 2004).

Hyperglycemia has been shown to inhibit autophagy-mediated degradation of cellular components and alter proteasomal activity (Queisser et al., 2010; Kobayashi et al., 2012). It is suggested that perturbations of PQC may be important factors in the development of diabetic cardiac failure. Indeed, therapeutic activation of autophagy in experimental diabetes type 1 and 2 models lead to improved cardiac function and reduced the incidence of cardiac abnormalities (Sciarretta et al., 2015). Both type 1 and type 2 diabetes mellitus have been shown to affect cellular mechanisms of PQC. Type 1 diabetes has been reported to decrease proteasomal activity but also increase the expression of proteasomal subunits (Powell et al., 2008; Li et al., 2017). In type 2 diabetes skeletal muscle showed increased expression of selected proteasomal subunits (Hwang et al., 2010). Whether autolysosomal activity is generally increased or impaired in type-1 and type-2 diabetes is still under debate (Mellor et al., 2011; Kobayashi et al., 2012; Xu et al., 2013). It was recently (Kanamori et al., 2015) reported that autophagy is increased in diabetic cardiomyopathy in type 1 diabetes, whereas it is reduced in hearts of patients with type 2 diabetes. Their conclusion of increased autophagy was partially based on findings of increased LC3-II and p62/sequestosome-1 level in a type 1 diabetes model. This observation was challenged by recent findings, showing elevated LC3-II and p62 protein levels also after pharmacological inhibition of autophagy in cultured cardiomyocytes (Müller et al., 2021). Apart from disunity concerning elevated or reduced autophagy, modification of PQC seems to be a general phenomenon in the setting of diabetes. However, the specific effects on sarcomere protein turnover particularly of titin and cardiomyocyte function have not been studied, to date. Diverging alterations in PQC in type -1 and type- 2 diabetes are likely to differentially affect the sarcomere and the titin filament and should be elucidated in future studies.

### 5.4 Mutations in Sarcomeric/Myofibrillar Proteins

Emerging evidence suggests that not only sarcomeric proteins but also non-filament proteins play an essential role in the development of various muscle diseases. A substantial overview of disease causing mutations in myofibrillar myopathy associated proteins has recently been given in a series of reviews [e.g., (Kley et al., 2016; Dorsch et al., 2019; Kellermayer et al., 2019; Rees et al., 2021)]. Therefore, we briefly highlight selected disease causing mutation in the titin sequence and other filamentous proteins.

#### 5.4.1 Titin Truncating and Missense Variants

Whole genome sequencing studies have revealed titin as an important human disease gene (Bang et al., 2001; Begay et al., 2015; Kellermayer et al., 2019). Due to the enormous size of the titin gene, diverse missense mutations have been observed at extremely high frequency (Karczewski et al., 2020). Because many of the missense mutations have also been found in control subjects and not only in cardiomyopathy patients, it was assumed that most of the mutations do not cause the disease (Thomson et al., 2019). Nonetheless, about 130 pathogenic *TTN* coding mutations were reported in relation to skeletal and/or cardiac myopathies (Chauveau et al., 2014; Savarese et al., 2016). Recessive truncation mutations adjacent to the C-terminal M-band were related to Salih myopathy that affects skeletal and cardiac muscle (Carmignac et al., 2007), while recessive mutations in centronuclear myopathy with potential cardiac involvement (Ceyhan-Birsoy et al., 2013) and other neuromuscular phenotypes were identified within the N-terminal region, and comprise compound heterozygosity of either two truncating mutations or a truncation and a missense mutation (Chauveau et al., 2013; Palmio et al., 2014).

The titin missense variant A178D was identified as the most likely cause of cardiac disease in a family affected by the uncommon autosomal dominant left ventricular non-compaction (LVNC) and dilated cardiomyopathy (DCM) (Hastings et al., 2016). In a mouse model carrying this mutation, the variant was sufficient to cause a mild phenotype of DCM (Jiang et al., 2021). The missense variant A178D causes disruption of the interaction between titin and telethonin (T-cap), leading to dissociation of telethonin from the Z-disc and cytosolic telethonin accumulation (Jiang et al., 2021). Subsequent depletion of cytosolic telethonin by the UPS leads to proteasome overload and functional impairment (Hastings et al., 2016).

**FIGURE 4:**
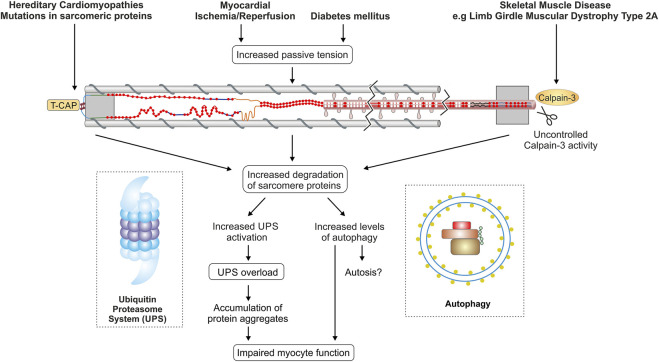
Titin and protein quality control in different disease settings. The figure summarizes currently discussed mechanisms of disturbed PQC affecting titin in cardiac and skeletal muscle diseases. TCAP, telethonin/titin-cap.

The most common form of cardiomyopathy is dilated Cardiomyopathy (DCM) with a prevalence of ∼1:250 (Hershberger et al., 2013; Schafer et al., 2017). Up to 25% of familial DCM cases are associated with heterozygous titin mutations, and many are titin truncating variants (*TTNtv*) (Herman et al., 2012; Roberts et al., 2015). Truncations in the constitutively expressed A-band region of titin, which stabilizes myosin filaments in the A-band, show the most obvious pathogenicity of *TTNtv* (Herman et al., 2012; Roberts et al., 2015; Schafer et al., 2017). Correction of truncated titin proteins using CRISPR/CAS technique results in functional recovery and appears to be a promising therapeutic target for the treatment of titin mutation-based cardiomyopathy (Fomin et al., 2021). Figure 4 summarizes alterations of PQC and titin in different disease settings.

#### 5.4.2 Mutations in Other Sarcomeric Proteins

Mutations in the *MYBPC3* gene coding for MyBP-C are the most common cause of familial hypertrophic cardiomyopathy (HCM) (Ho et al., 2018). Most *MYBPC3* variants are frameshift, nonsense or splice variants resulting in premature stop codons. Truncated transcripts are targeted for degradation by nonsense mediated RNA decay and result in reduced levels of MyBP-C protein (Helms et al., 2020). In heart tissues of HCM patients MyBP-C protein level was reduced by approximately 40% and lower synthesis rates are compensated by diminished MyBP-C degradation (Marston et al., 2009; O'Leary et al., 2019; Helms et al., 2020). Non-truncating variants account for up to 15% and a subset of domain specific variants causes loss of function due to failure of myofilament incorporation (Helms et al., 2020). Heat shock cognate 70 kDa (HSC70) was identified as a central player in the turnover of the wild type as well as the mutated MyBP-C (Glazier et al., 2018).

Mutations in *FLNC*, the gene coding for filamin C, causes myofibrillar myopathy (filaminopathy) characterized by disintegration of myofibrils predominantly at the Z-disc and massive protein aggregates in cytosolic deposits within the muscle fibers (Kley et al., 2013). Components of the CASA complex HSPA8, HSPB8 and SQSTM1/p62, were accumulated in the aggregates while BAG3 and STUB1 localizes around the deposits. These results indicate that abnormal muscle fibers compensate the massive protein aggregation with a strongly increased expression of proteins involved in proteasomal and autophagic degradation (Kley et al., 2013).

The scientific community is just beginning to recognize the absolute number of involved proteins and disease causing mutations, triggered by new RNA and DNA sequencing technologies. More research is required to uncover the pathomechanisms by which mutations in myofibrillar and non-myofibrillar proteins induce myopathies. Future studies will uncover new exciting insights with potentials for therapeutic interventions.

## 6 Conclusion

In summary, the turnover of titin filaments and other sarcomere proteins depends on a whole range of PQC mechanisms. Loss of the physiological balance of synthesis and degradation under pathological conditions could lead to either insufficient or even excessive turnover of sarcomere proteins and titin. Decreased titin turnover due to impaired autophagy or reduced proteasomal activity results in partially damaged titin filaments within the sarcomere, which then compromise the contractile and elastic properties of the sarcomere (Figure 4). In contrast, abnormally increased degradation possibly leads to turnover of the still functional and not only the heavily stressed and worn titin filaments. More detailed knowledge of the finely tuned ubiquitination and turnover process of sarcomere proteins is necessary to understand how pathological changes in these processes affect sarcomere function and cardiac and skeletal muscle properties. In the future, the targeted use/modification of PQC components may represent a promising option for therapeutic intervention.
